# Social Odour Perception and Stress Responses in Women’s Quality of Partner Relationship and Attachment Style

**DOI:** 10.3390/bs13030239

**Published:** 2023-03-09

**Authors:** Giulia Piraino, Omar Carlo Gioacchino Gelo, Andrea Schito, Lydia Giménez-Llort, Sara Invitto

**Affiliations:** 1 INSPIRE LAB, Laboratory of Cognitive and Psychophysiological Olfactory Processes, DiSTeBA, University of Salento, 73100 Lecce, Italy; 2 Department of Human and Social Sciences, University of Salento, 73100 Lecce, Italy; 3 Faculty of Psychotherapy Science, Sigmund Freud University Vienna, 1020 Vienna, Austria; 4 Department of Psychiatry and Forensic Medicine, School of Medicine, Universitat Autònoma de Barcelona, 08193 Barcelona, Spain; 5 Institut de Neurociències, Universitat Autònoma de Barcelona, 08193 Barcelona, Spain

**Keywords:** intimate partner violence, stress, social odour perception, attachment style, emotional regulation, women

## Abstract

The perception of body and social odours (SOP) is crucial for interpersonal chemosensory signalling and mate choice, yet little is known about the role of the SOP on the quality of partnerships and the attachment style. The aim of this study was to investigate the role of the SOP in women’s stress responses by considering the role of biopsychosocial variables in the quality of interpersonal relationships (also considering intimate partner violence). In total, 253 women filled out an online survey that included a series of questionnaires to investigate self-perceived stress (PSS), emotional regulation (ERQ), olfactory social assessment (SOS), quality of partnership (RRQ), attachment style (RQ), and the Conflict Tactile Scale 2 (CTS-2). The main results highlight that a high awareness of social odours correlates with a good quality of relationship and with an emotional regulation capacity; the PSS correlates negatively with the ERQ (i.e., as the PSS increases, the ERQ decreases). The level of IPV predicts an interpersonal style characterized by a low desire to develop meaningful relationships but with a tendency to depend on and trust another. The idea of being hurt by the other is not central in women who experience this type of relationship. The study’s main conclusion is that social odour perception is important for emotional regulation and in partner relationships.

## 1. Introduction

When exposed to a stressor, an organism develops nonspecific reactions that are independent of the type of stimulus and modulate dysfunctional changes in the organism itself affecting the perceptual systems and the organism’s quality of life [1,2,3,4].

Women are exposed to stressful conditions more frequently in relation to the gender care roles with which they are culturally associated [5]. Several studies point out that women also experience higher levels of chronic stress than men in terms of intensity [6,7,8,9]. This could be particularly true for clinical psychology and the challenges of the current uncertain times, such as the COVID-19 pandemic [10]. The latter has introduced a unique set of challenges and stressors that have negatively affected mental health and wellness [11].

In this framework, the olfactory information process could play a pivotal role. Both cognitive and psychophysiological studies suggest that the sense of smell has relevant modulatory effects on a wide spectrum of functions, such as memory, emotions, and social behaviour [3,12]. Indeed, the olfactory system shares close subcortical connections with the limbic system, which also suggests that chemical stimuli can be directly involved with stress reactions [13,14]. For instance, people with post-traumatic stress disorder often report that smells associated with trauma trigger painful memories and states of fear, anguish, and terror, as if what happened in the past was imminent in the present [15]. Olfaction is a mechanism involved in the creation of significant and essential bonds for the individual and his/her survival as well as in triggering specific behaviours [16,17,18]. Moreover, it is well known that body and social odours are crucial for interpersonal chemosensory signalling and human communication [19,20] and also for mate choice [20]. A partner’s body odour is associated with security, since it seems to reduce the skin conductance response and the unpleasantness of a stressful stimulus, especially in secure-attachment individuals [21]. In fact, olfaction is related to romantic relationship maintenance and body odours, particularly in women, which could be fundamental for evoking the attachment system [22]. Moreover, Croy and colleagues [23] reported that anosmic women reported significantly higher degrees of romantic relationship insecurity. These studies suggest that greater attachment insecurity is related to reduced self-reported olfactory perception.

Therefore, an olfactory impairment is related to a reduced capacity to interpret nonverbal communication [22]. Thus, olfactory perception could have an impact on partnership communication, nonverbal communication, and emotional regulation.

Therefore, it is clear that biopsychosocial variables in relation to interpersonal dynamics are important moderators in the relationship between stress and health [24,25]. A phenomenon capable of highlighting this relationship could be intimate partner violence (IPV), which is defined by the Centers for Disease Control and Prevention as ‘physical violence, sexual assault, stalking and psychological assault (including coercive acts) by a current or previous intimate partner’ [26]. The IPV experience is highly stressful for the victims [27], triggering relevant consequences on their functioning at the behavioural [28], neuropsychological and cognitive [29,30,31], emotional [32], and physiological [33,34,35,36] levels. However, there is not always a correspondence between biological reactions to stress and their subjective perception, because the latter is filtered by factors such as self-awareness and the subjective meaning given to what is experienced. According to cognitive psychology, the ways in which we interact and communicate are determined by the way we interpret reality, our way of thinking, and our opinions and expectations. These are the product of the interpersonal experiences in which we have lived.

This perspective is based on the role played by internal operating models [37], i.e., the representations about the outcome of the relationship, about the idea that a person has of himself/herself and of the other, and in relation to the behavioural strategies developed.

Starting from these premises, this study aims to investigate the role of social odour perception linked to the stress responses in women by considering the role of biopsychosocial variables in the quality of interpersonal relationships. In particular, we address how a condition of different levels of persistence and chronicity in domestic conflictual relations can have specific indicators also in social odour perception and attachment style.

## 2. Materials and Methods

### 2.1. Participants

An online survey was completed by 253 women (mean age = 35.99, s.d. = 5.45). The sample can represent the entire female population with a confidence level of 95% and a margin of error of 6.16%. Subjects were recruited in the period from 30 January to 30 March 2022; all of them participated in the research by following a link presented through social networking services (e.g., Facebook, Cambridge, MA, USA and LinkedIn, Microsoft^®^, Albuquerque, NM, USA). Of the sample, 71.9% of the women were engaged in a partner relationship that had lasted for more than three years. In this research, we did not consider women who had used centres against domestic violence. We assume that women using these centres have already experienced high levels of violence and stress, have taken charge and undergone a specific psychotherapy for this type of violence, and had access to legal, social support, etc.; these variables could modify the outcome of our research.

The study was conducted in accordance with the Declaration of Helsinki. The participants did not receive any financial compensation for their participation. All data were anonymous, unidentifiable, and numerically coded for statistical purposes. All participants read and signed the informed consent form before starting the questionnaire, as required by the Helsinki Declaration. They also signed off on the processing of personal data and respect for privacy (law no. 675, 676 of 31 December 1996, Official Gazette of 8 January 1997, art. 7 of Legislative Decree 30 June 2003, no. 196 and EU Privacy Regulation 2016/679, General Data Protection Regulation—GDPR); all data (including sensitive data) were treated strictly anonymously. The research (protocol code 01-2022) was approved by the Institutional Review Board of Biological and Environmental Sciences and Technologies (DiSTeBA) of the University of Salento, Lecce Italy, 20 January 2022.

### 2.2. Scales and Questionnaires

The survey was divided into two sections: the first one included personal data and social questions; the second section included a series of behavioural questionnaires investigating the cognitive, emotional, and perceptive functioning of women. The specific tests administered were the Perceived Stress Scale (PSS) [38], the Social Odor Scale (SOS) [39], the Romance Qualities Scale (RQS) [40], the Relationship Questionnaire (RQ) [41], the Emotion Regulation Questionnaire (ERQ) [42], and the Conflict Tactics Scale 2 (CTS 2) [43]) for assessing conflict behaviour in couples and identifying IPV in the female population.

#### 2.2.1. Violence Chronicity

The group was divided into levels using the scores obtained in the CTS2. The prevalence score made it possible to highlight whether one or more acts listed in the scale were used in the past year. This score was obtained by creating a dichotomous scale, attributing a score of 1 or 0. A score of 1 indicated one or more acts of violence in the past year; a score of 0 indicated that there were no violent acts in the past year. This method assigned a score of 1 for any subject who reported one or more instances of any of the acts in the scale. The prevalence score is appropriate for the psychological, physical, and sexual assault scales, as well as for the injury scale, because the key issue is the percent of the population in which an assault or injury occurred during the referent period. The chronicity score, on the other hand, is the sum of the number of times each act in the scale was experienced by those who experienced at least one of the acts in the scale. Therefore, the group was divided into three levels of chronicity: 0 (i.e., 0,) 1 (i.e., scores 1 ÷ 9), and 2 (i.e.g, scores > 10).

#### 2.2.2. Attachment Style

The attachment style was determined through the Relationship Questionnaire (RQ), which involved four items describing four general relationship styles. It asked the women to select the one which they felt best described or was closest to their style of relating to people. Subsequently, for each style described, the participant was asked to assign a score on a Likert scale, from 1 to 7, where 1 indicated ‘very different from me’ and 7 indicated ‘very similar to me’.

#### 2.2.3. Emotional Regulation Capacity

The emotional regulation capacity was investigated through the Emotional Regulation Questionnaire (ERQ), with 10 items investigating emotional experience (i.e., what the woman feels) and the expression of emotional experience (i.e., the way in which emotions are shown). Each item asked the participants to score on a Likert scale from 1 to 7, where 1 indicated ‘strongly disagree’ and 7 ‘totally agree’.

#### 2.2.4. Perceived Stress

Perceived stress was assessed through the Perceived Stress Scale (PSS), a 10-item questionnaire, requesting a score from 0 to 4 for each item, where 0 stood for ‘never’, 1 indicated ‘almost never’, 2 stood for ‘sometimes’, 3 indicated ‘quite often’, and 4 stood for ‘very often’. The items allowed us to ascertain the level at which people who responded to the test found their lives to be unpredictable, uncontrollable, or overloaded. The scale also contained a series of direct questions about current levels of perceived stress.

#### 2.2.5. Quality of the Relationship

The quality of the relationship with a partner was investigated through the Romance Qualities Scale (RQS), a 22-item scale that represented the five indicators of the quality of the relationship: closeness, conflict, compassion, help, and security. The score attributed to each item was expressed on a Likert scale, from 1 to 5, where 1 stood for ‘absolutely false’ and 5 represented ‘absolutely true’.

#### 2.2.6. Recognition of Social Odour

With regard to the role of smell, the Social Odor Scale (SOS), a 12-item scale to investigate the awareness of different social odours, was used.

### 2.3. Statistical Analysis

IBM SPSS Statistics software (IBM Corporation, Armonk, NY, USA) version 27.0.1 and JASP 0.16.1 open-access software (University of Amsterdam, Amsterdam, The Netherlands) were used for statistical data analysis. A one-way MANOVA design was used in order to explore the influence of the chronicity of violence (independent variable, 3 levels) on the scores obtained in all the scales and questionnaires of the survey. Therefore, ten dependent variables were used, and they were represented by the total scores of the SOS, PSS, RQS, and CTS2, the total scores of two subscales of the ERQ (i.e., suppression (ERQ_Sup) and revaluation (ERQ_Rev)), the scores of each item of the RQ (i.e., the secure, fearful, preoccupied, and dismissing attachment styles). In addition, a correlation analysis and a regression were performed between all tests by Pearson’s coefficient, and the *p*-values were adjusted for multiple comparisons with Bonferroni correction. The statistical significance was set at *p* ≤ 0.05 for all analyses.

## 3. Results

From the total sample of 253 women, about half of them (136 of 253) were at a level 0 of violence chronicity; 30% (76 of 253) were at level 1, and 15% (39 of 253) were at level 2.

The MANOVA was not significant (*p* = 0.428).

Concerning the correlation analysis (Table 1), a significant relationship was found between the ability to recognize social odour (SOS) and the quality of the intimate relationship (RQS) (r = 0.188; *p* = 0.003), as well as between the SOS and the emotional regulation capacity through revaluation (ERQ_Rev) (r = 0.193; *p* = 0.002). The highest correlation was found between perceived stress (PSS) and the ERQ_Rev (r = −0.265; *p* < 0.001).

**Figure 1:**
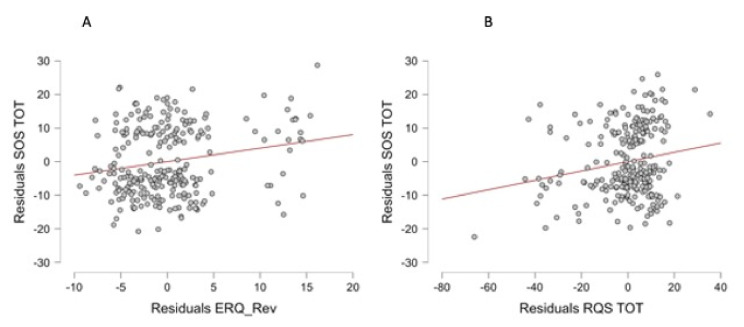
The relationship between the average SOS and (**A**) the ERQ_Rev and (**B**) the RQS TOT. Each data point represents one participant. The regression line is indicated by the intersected red line.

A linear regression analysis was also performed considering the SOS as the dependent variable and the levels of chronicity as the factor. The linear regression highlighted significant results (F = 2.432; *p* = 0.005); in particular, the model showed a significant value for the RQS TOT (t = 2.915; *p* = 0.004) and ERQ Rev (t = 3.114; *p* = 0.002) (see Figure 1).

**Figure 2:**
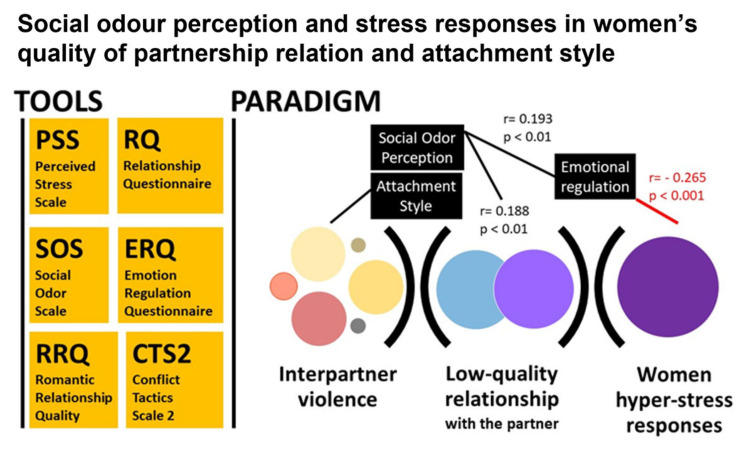
Integrative Map of the role of social odour perception as a modulator of women’s stress responses in the quality of the partner relationship and attachment style, as found in Figure 1 and Table 1.

As summarized in Figure 2, these results highlighted how the experience of intimate violence predicted an interpersonal style (attachment) characterized by a low desire to develop meaningful relationships but a tendency to depend on others and to trust the other. The idea of being hurt by the other was not central in women who experienced this type of partner relationship.

Furthermore, the results showed how awareness of social odours correlated with a good quality of relationship with the partner and with an adequate emotional regulation capacity characterized by a revaluation of the activating situations. Perceived stress, on the other hand, correlated negatively with the same emotional regulation ability; as perceived stress increased, the emotional regulation ability decreased.

## 4. Discussion and Conclusions

While biological studies have almost exclusively conceptualized IPV as a predictor of biological outcomes, psychological studies highlight the potential bidirectionality of the association between IPV and stress: IPV can predict stress, but stress can also increase the risk of the onset or chronicity of IPV. The psychological literature provides strong evidence that IPV is associated with psychological distress, and the few longitudinal studies [44] further suggest that psychological distress follows new cases of IPV. Large-scale integrative studies are needed that use prospective study designs more closely mapping how IPV affects victims both biologically and psychologically and how these biopsychological changes, in turn, affect victims’ health over time.

A woman experiencing an abusive relationship as a traumatic and chronic experience seems to lead the victim to an emotional, behavioural, and cognitive normalization of what she has experienced. This normalization is understandable as a strategy to deal with traumatic events capable of making the woman perceive a threat to her own self to her own life. Each individual has a typical body odour that in terms of physical features reflects stable personal characteristics or transient events. These odours are perceived by other people and influence their reactions: they become social odours capable of influencing behaviour.

A behavioural study of the olfactory component, specifically in relation to the awareness of body odours, has made it possible to add important information to the analysis of the relationship between smell and stress in partner relationships.

The literature suggests that another person’s scent can activate memories, trigger emotions, and arouse romantic attraction as well as impact both psychological and physiological reactions to stress [45].

One study found that exposure to a stranger’s odour was rated as being more intense and less pleasant than experiencing a friend’s odour, and a stranger’s odour activated cortical regions associated with viewing threatening stimuli [46]. Thus, it is possible that detecting a stranger’s odour could be a unique signal of physical proximity to a potentially dangerous individual, triggering an increase in perceived stress and/or mobilizing the body’s physical resources for an uncertain event. This mobilization could activate the hypothalamic–pituitary–adrenal axis, resulting in increased cortisol levels.

The observation of a correlation between the awareness of social odours and the quality of the relationship with a partner as well as with a functional emotional regulation shows how the odours produced by the human body are essential in interpersonal relationships and in the selection of one’s partner, in line with the literature in the field [19,20].

The present study suggests that olfactory perception may have effects on emotion and affect, but the reverse may also be possible; that is, emotions, affective states, and atypical conditions in the emotional processing might influence odour awareness. Therefore, a low awareness of social odour perception, caused by a condition of chronic stress such as domestic violence, could lead to greater difficulty in recognizing danger signals within a couple’s relationship.

The results highlight specific characteristics in the functioning of women who experience a conflictual relation: living in a violent relationship determines a normalization of what one has suffered, implying interpersonal modalities characterized by a difficulty in recognizing the danger as it is cognitively normalized and physiologically coded incorrectly. This condition is consistent with previous work in the literature that analysed the functioning of women through electrophysiological data [31].

This study had some limitations. First, the questionnaire was performed online, limiting experimental control over the respondents. Furthermore, the scores reflect self-perceived behaviour rather than the behaviour itself and thus could be influenced by response bias, a problem with all assessments involving self-reports. The hope is that these data will soon be integrated and correlated with EEG measurements to overcome the major limitation of the current results.

Furthermore, further research can be developed by including women who have used anti-violence centres, to evaluate whether this model recurs even in a situation where the problem is addressed. The connection between smell and evolutionary adaptive responses in this case appears to be strictly relevant because smell, which is apparently held in second order with respect to other senses, turns out to be absolutely sensitive to stressful and affective aspects, also correlating itself to aspects of emotional regulation. This could be especially important in the therapeutic and recovery phase of healthy psychophysiological and relational dimensions.

## Figures and Tables

**Table 1:** The correlation analysis between the perceived stress (PSS), social odours (SOS), romantic relationship quality (RQS), and emotional regulation capacity through revaluation (ERQ_Rev).

Variable		SOS	PSS
**RQS**	Pearson’s r	0.188	−0.018
	*p*-value	0.003	0.780
	*p*-correction (Bonferroni)	0.036	1.0
**ERQ_Rev**	Pearson’s r	0.193	−0.265
	*p*-value	0.002	<0.001
	*p*-correction (Bonferroni)	0.024	0.012

## Data Availability

Data are available by writing to giulia.piraino@unisalento.it.
